# Abdominal obesity and gastroesophageal cancer risk: systematic review and meta-analysis of prospective studies

**DOI:** 10.1042/BSR20160474

**Published:** 2017-05-11

**Authors:** Xuan Du, Khemayanto Hidayat, Bi-Min Shi

**Affiliations:** Department of Endocrinology and Metabolism, The First Affiliated Hospital of Soochow University, Suzhou 215006, China

**Keywords:** abdominal obesity, central obesity, esophageal cancer, gastric cancer, waist circumference, waist to hip ratio

## Abstract

To systematically and quantitatively review the relation of abdominal obesity, as measured by waist circumference (WC) and waist to hip ratio (WHR), to total gastroesophageal cancer, gastric cancer (GC), and esophageal cancer. PubMed and Web of Science databases were searched for studies assessing the association between abdominal obesity and gastroesophageal cancer (GC and/or esophageal cancer) up to August 2016. A random-effect model was used to calculate the summary relative risks (RRs) and 95% confidence intervals (CIs). Seven prospective cohort studies – one publication included two separate cohorts – from six publications were included in the final analysis. A total of 2130 gastroesophageal cancer cases diagnosed amongst 913182 participants. Higher WC and WHR were significantly associated with increased risk of total gastroesophageal cancer (WC: RR 1.68, 95% CI: 1.38, 2.04; WHR: RR 1.49, 95% CI: 1.19, 1.88), GC (WC: RR 1.48, 95% CI: 1.24, 1.78; WHR: 1.33, 95% CI: 1.04, 1.70), and esophageal cancer (WC: RR 2.06, 95% CI: 1.30, 3.24; WHR: RR 1.99, 95% CI: 1.05, 3.75).Findings from our subgroup analyses showed non-significant positive associations between gastric non-cardia adenocarcinoma (GNCA) and both measures of abdominal adiposity, while gastric cardia adenocarcinoma (GCA) was positively associated with WC but not with WHR. On analysis restricted to studies that adjusted for body mass index (BMI), WC was positively associated with GC and esophageal cancer, whereas WHR was positively associated with risk of GC only. Although limited, the findings from our meta-analysis suggest the potential role of abdominal obesity in the etiology of gastric and esophageal cancers.

## Introduction

Globally, esophageal cancer ranks eighth for cancer incidence and sixth for cancer death, while gastric cancer (GC) ranks fourth and second, respectively [1]. There is mounting evidence that obesity increases the risk of certain types of cancers, including post-menopausal breast cancer, colorectal, endometrial, kidney, and pancreatic cancers [2–7]. Obesity may have also contributed to the recent rise in gastric cardia carcinoma and esophageal adenocarcinoma (EAC) incidence over the past decades because the prevalence of obesity has increased dramatically at an accelerating and alarming rate during approximately the same time period [8,9]. According to World Cancer Research Fund/American Institute for Cancer Research (WCRF/AICR) report from 2016, the association of general obesity, as measured by the body mass index (BMI), with esophageal cancer has been judged convincing by the panel [10], whereas the evidence for an association with GC has remained less conclusive [11]. In spite of its wide use, BMI as a measure of obesity is not always accurate and is controversial [12,13]. More importantly, neither BMI differentiates between excess body weight due to high levels of fat mass or muscle mass, nor does it permit assessment of the distribution of fat mass.

During the past few years, evidence from several observational studies has shown that body fat distribution, particularly abdominal obesity, may better predict risk of several chronic diseases and mortality than general obesity (BMI) [14–18]. Moreover, regardless of the definition used, abdominal obesity, rather than general obesity, is considered to be one of the key features of metabolic syndrome [19]. Furthermore, abdominal obesity may pose higher threat to health than general obesity, as intra-abdominal fat cells tend to be more detrimental and metabolically active than the other fat in the body [20,21]. In addition, intra-abdominal fat has been hypothesised to be biologically different from fat in other areas with regard to tumor angiogenesis and cell proliferation [22,23]. Although general obesity has emerged as a potential risk factor for gastroesophageal cancer, but the association between abdominal obesity and gastroesophageal cancer is still poorly understood, partly because of sparse evidence from prospective studies available at that time, which limited the strength of the conclusions [24–29]. Given these considerations, providing clear evidence regarding potentially detrimental effect of abdominal obesity on gastroesophageal cancer would be important for clinical interventions, including weight loss program or weight management program and gastroesophageal cancer screening guidelines for obese individuals. Therefore, to better understand the relationship between abdominal obesity and gastroesophageal cancer, we performed a comprehensive systematic review and meta-analysis of prospective studies that examined the association between abdominal obesity and risk of these malignancies.

## Materials and methods

### Search strategy

This meta-analysis was planned, conducted, and reported according to ‘Meta-analysis of Observational Studies in Epidemiology (MOOSE) group’ guidelines [30]. PubMed and Web of Science databases were searched for studies assessing the association between abdominal obesity and gastroesophageal cancer up to August 2016. The following search terms were employed to retrieve the relevant literature in the databases: (adiposity OR body size OR anthropometric OR abdominal obesity OR central obesity OR obese OR abdominal adiposity OR obesity OR body composition OR body fat distribution OR body fat patterning OR retroperitoneal fat OR visceral fat OR abdominal fat OR intra-abdominal fat OR waist to hip ratio OR waist-hip ratio OR waist circumference OR girth circumference OR abdominal adiposity measures OR adiposity measures) AND (stomach cancer OR cancer of stomach OR gastric cancer OR gastric carcinoma OR gastric adenocarcinoma OR gastric cardia carcinoma OR gastric non-cardia carcinoma OR gastric neoplasm OR gastric tumor OR tumor of stomach OR esophageal cancer OR cancer of esophageal OR esophageal carcinoma OR esophageal adenocarcinoma OR esophageal tumor OR tumor of esophagus) AND (cohort OR prospective OR follow-up OR follow up OR observational study). The search strategy had no language, publication date, or publication-type restriction. In addition, the reference lists of retrieved full publications and previous meta-analysis were reviewed to complement the search and to identify relevant studies that were missed during electronic database search.

### Study selection

To be included in this meta-analysis, the studies had to meet the following inclusion criteria: (i) the study design was a prospective study (including prospective cohort study, nested case-control study, and case-cohort study); (ii) investigated the association between WC and/or WHR and gastroesophageal cancer (GC and/or esophageal cancer); (iii) relative risks (RRs), hazard ratios (HRs), or odds ratio (ORs) with 95% confidence intervals (CIs) were available. Accordingly, retrospective studies or studies on gastroesophageal cancer mortality, or recurrence were excluded. If multiple publications from the same study were identified, the publication containing the largest number of cases and most applicable information was selected.

### Data extraction and quality assessment

Using a standardised data-collection form, the following data were abstracted from each study: the first author’s last name, publication year, country, study population, duration of follow-up, number of participants, number of cases, ascertainment of adiposity, measures of abdominal adiposity, most fully adjusted risk estimates with their corresponding 95% CIs for each category of abdominal adiposity measures, statistical adjustment for potential confounding factors, and outcome assessed. If multiple RRs of the association were available, we extracted RRs with their corresponding 95% CIs from the models that reflected the maximum extent of adjustment for potentially confounding variables. When studies provided specific risk estimates (i.e. anatomic subtypes of gastric adenocarcinoma), we extracted all of them and used the data in subgroup analyses. The study quality was assessed using the 9-star Newcastle-Ottawa Scale (NOS) [31], in which each study was judged based on the selection of the study groups (representativeness, selection of non-exposed cohort, ascertainment of exposure, no disease at start of study), the comparability of the groups, and three for the quality of the outcome (assessment of outcome, length of follow-up, and adequacy of follow-up). Studies with NOS values of six or higher were considered moderate to high quality studies and those with a NOS value of less than six were regarded as low quality. Two investigators (X.D. and K.H.) participated in literature search, study selection, data extraction, and quality assessment independently. Any discrepancies regarding inclusion were solved through group discussion, with input from the senior investigator (B.-M.S.).

### Statistical analysis

RR was chosen as the common measure of association across the present study. Due to the rarity of cancer in the general population, HR and OR were directly considered as RR. DerSimonian and Laird [32] random-effects model was used to calculate the summary risk estimates. The degree of heterogeneity in the relationship between abdominal adiposity and gastroesophageal cancer across studies was assessed using *Q* and *I*^2^ statistics. For the *Q* statistic, *P*<0.1 was considered statistically significant; and for the *I*^2^ statistic, the following conventional cut-off points were used: <25% (low heterogeneity), 25–50% (moderate heterogeneity) and >75% (severe heterogeneity). Both Begg’s rank correlation test and Egger’s linear regression test were performed to investigate potential publication bias [33]. If evidence of publication bias was observed, the trim-and-fill method was applied to correct the bias [34]. To explore potential sources of heterogeneity, subgroup analyses was performed according to sites of cancer (GC or esophageal cancer) and anatomic subtypes of gastric adenocarcinoma (gastric cardia adenocarcinoma (GCA) or gastric non-cardia adenocarcinoma (GNCA)). To investigate the affects of individual studies on the overall results, we also performed a sensitivity analysis by omitting one study in each turn, while pooling results from the remainder. All statistical analyses were performed using STATA software, version 11.0 (STATA Corp., College Station, TX, U.S.A.). All *P*-values were two-sided and the level of significance was at <0.05, unless explicitly stated.

## Results

### Literature search and study characteristics

**Figure 1:**
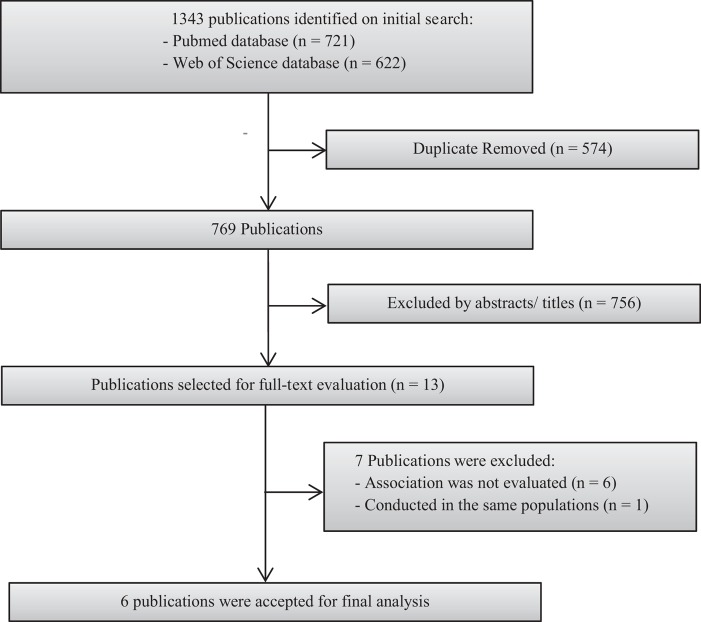
Flow chart of study selection

Figure 1 presents a flow chart showing the study selection process. We initially identified 1343 potential articles from PubMed and Web of Science databases; most were excluded because they were not prospective studies or because the exposure or outcome was not relevant to our analysis, leaving 13 potentially eligible papers for full-text review. Previous study on EAC by Steffen et al. [35] was excluded. Instead, we included the updated study [27], which also investigated the association between abdominal obesity and GC. Furthermore, six studies [36–41] were excluded because the risk estimate for the association of interest was not available. Finally, seven prospective cohort studies [24–29] – one publication [28] included two separate cohorts – from six publications were included in the final analysis. The characteristics of the included studies are summarised and listed in Table 1. These studies were published between 2005 and 2016. All the included studies had a prospective cohort design. A total of 2130 gastroesophageal cancer cases were diagnosed amongst 913182 participants. Two prospective cohort studies were conducted in the United States [25,26] and Norway [28], and one each in Australia [24], multiple European countries [27], and China [29]. Regarding the sex of the participants, one study [29] evaluated only women and the remaining six [24–28] included both sexes. The length of follow-up ranged from 6.2 to 15.1 years. Individual studies adjusted for a wide range of potential confounding factors, such as age, BMI, and smoking. The details of quality assessment according to the nine-star NOS are presented in the online Supplementary Table. The qualities of the studies ranged from low to high (mean NOS score: 7.16, range: 5–8).

**Table 1:** Prospective studies of abdominal obesity and gastroesophageal cancer

References (country)	Study population (age)	Duration of follow-up (years)	Sample size (gastroesophageal cancer cases)	Ascertainment of adiposity	Measure of adiposity	Categories, highest compared with lowest (measurement unit)	Adjusted RR (95% CI)	Adjustments
MacInnis et al., 2005 (Australia) [24]	Men and women (27–75 years)	11.3	41295 (98)	Trained	WC	Males: ≥102 cm compared with <94 cm; females: ≥88 cm compared with <80 cm	LE and GCA: 2.9 (1.2, 6.9); GNCA: 1.1 (0.6, 2.0)	Sex, country of birth, highest level of education and physical activity
					WHR	Males: 0.95 compared with <0.90; females: 0.80 compared with <0.75	LE and GCA: 2.1 (0.8, 5.5); GNCA: 0.9 (0.5, 1.7)	
O’Doherty et al., 2012 (U.S.A.) [25]	Men and women (50–71 years)	9	218854 (569)	Self-measured	WC^1^	Q4 compared with Q1	EAC: 2.03 (1.21, 3.39); GCA: 1.98 (1.11, 3.53); GNCA: 1.46 (0.71, 3.03)	Age, sex, total energy, antacid use, aspirin use, non-steroidal anti-inflammatory drug use, marital status, diabetes, cigarette smoking, education, ethnicity, alcohol consumption, physical activity, red and white meat intake, and fruit and vegetable intake
					WHR^2^	Q4 compared with Q1	EAC: 1.47 (0.99, 2.18); GCA: 1.08 (0.71, 1.63); GNCA: 1.46 (0.86, 2.48)	
Hardikar et al., 2013 (U.S.A.) [26]	Barrett’s esophagus patients (30 to ≥75 years)	6.2	411 (45)	Trained	WHR	Q4 compared with Q1	1.48 (0.60, 3.61)	Age, gender, NSAIDs use, and smoking status
Steffen et al., 2015 (European countries) [27]	Men and women (25–70 years)	11	391456 (541)	Trained	WC	Q5 compared with Q1	EAC: 3.76 (1.72, 8.22); GCA: 1.91 (1.09, 3.37); GNCA: 1.25 (0.75, 2.08)	BMI, sex, education, smoking habits, alcohol consumption at recruitment and amount of alcohol, physical activity and intake of red and processed meat, vegetables, citrus and non-citrus
					WHR	Q5 compared with Q1	EAC: 4.05 (1.85, 8.87); GCA: 1.95 (1.12, 3.38); GNCA: 2.05 (1.19, 3.52)	
Lin et al., 2015 (Norway) [28]	Men and women from the Cohort of Norway and the third Nord-Trøndelag Health Study (≥20 years)	10.2	192903 (499)	Trained	WC	Men: ≥94 cm compared with <94 cm; women: ≥80 cm compared with <80 cm	EAC: 2.48 (1.27, 4.85); ESCC^3^: 1.19 (0.71, 2.00); GC: 1.47 (1.14, 1.90)	Age, sex, BMI, education, smoking status, and family cancer history
Liu et al., 2015 (China) [29]	Shanghai women (40–70)	15.1	68253 (378)	Trained	WHR	>0.85 compared with ≤0.77	GC: 1.12 (0.79, 1.6)	Education, total energy intake, total vegetable and fruit intake, total meat intake, leisure-time physical activity, alcohol consumption, menopausal status, spouse smoking exposure, parity, family history of cancer, and additionally adjusted for BMI

ESCC, esophageal squamous cell carcinoma; LE, lower esophagus; NSAIDs, non-steroidal anti-inflammatory drugs; Q, quantile

^1^Additionally adjusted for hip circumference.

^2^Additionally adjusted for BMI.

^3^Additionally adjusted for alcohol intake.

### WC

**Figure 2:**
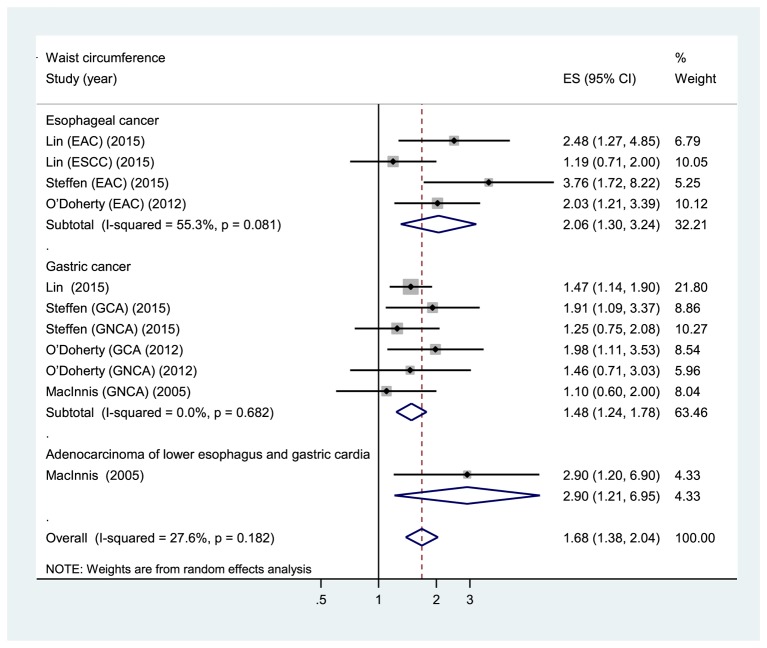
Forest plot of highest compared with lowest category of WC and gastroesophageal cancer risk. ESCC: esophageal squamous cell carcinoma.

**Figure 3:**
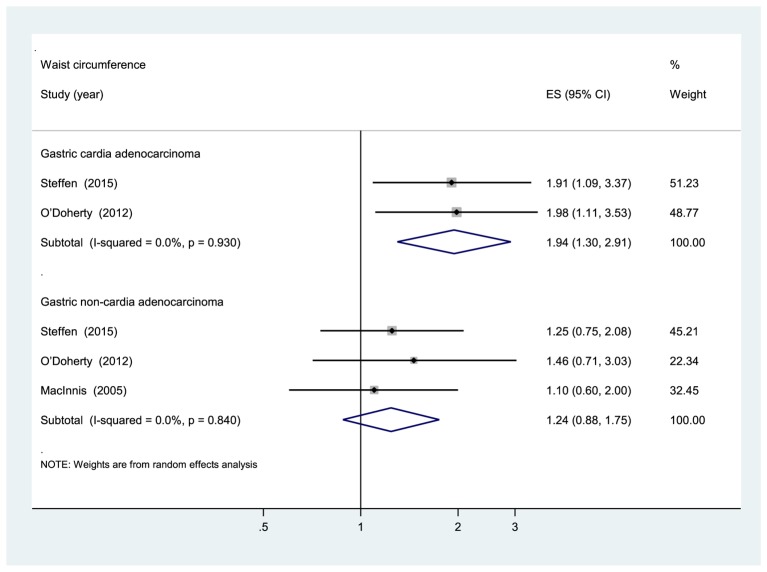
Subgroup analyses according to anatomic subtypes of GC (WC).

Five prospective cohort studies [24,25,27,28] were eligible for the analysis of WC. Comparison of the highest category of WC with the lowest category revealed significant associations between higher WC and increased risk of total gastroesophageal cancer (RR: 1.68, 95% CI: 1.38, 2.04), GC (RR: 1.48, 95% CI: 1.24, 1.78), and esophageal cancer (RR: 2.06, 95% CI: 1.30, 3.24) (Figure 2). The evidence of moderate heterogeneity were observed for total gastroesophageal cancer (*I*^2^ =27.6%, *P*=0.182), as well as for esophageal cancer (*I*^2^ =55.3%, *P*=0.081). On the other hand, no evidence of heterogeneity for GC (*I*^2^ =0%, *P*=0.682). Higher WC was associated with an increased risk of GCA (RR: 1.94, 95% CI: 1.30, 2.91; *I*^2^ =0%, *P*=0.930) but not with GNCA (RR: 1.24, 95% CI: 0.88, 1.75; *I*^2^ =0%, *P*=0.840) (Figure 3). Analysis restricted to two studies [27,28] that adjusted for BMI yielded RRs of 1.48 (95% CI: 1.20, 1.83; *I*^2^ =0%, *P*=0.547) and 2.13 (95% CI: 1.07, 4.22; *I*^2^ =70%, *P*=0.036) for GC and esophageal cancer, respectively. The sensitivity analyses that omitted one study at a time and calculated the combined RR for the remaining studies yielded consistent results. No evidence of publication bias was observed across studies (Begg, *P*>0.1; Egger, *P*>0.1).

### WHR

**Figure 4:**
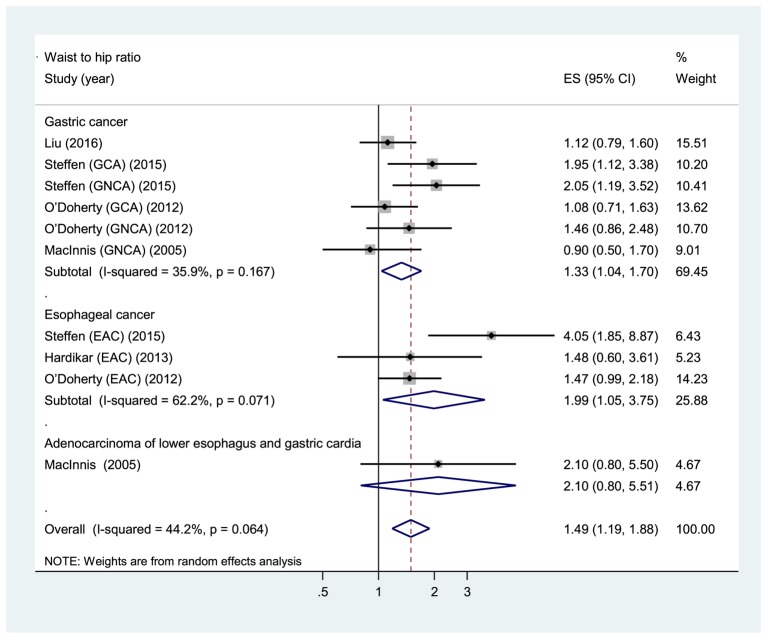
Forest plot of highest versus lowest category of WHR and gastroesophageal cancer risk.

**Figure 5:**
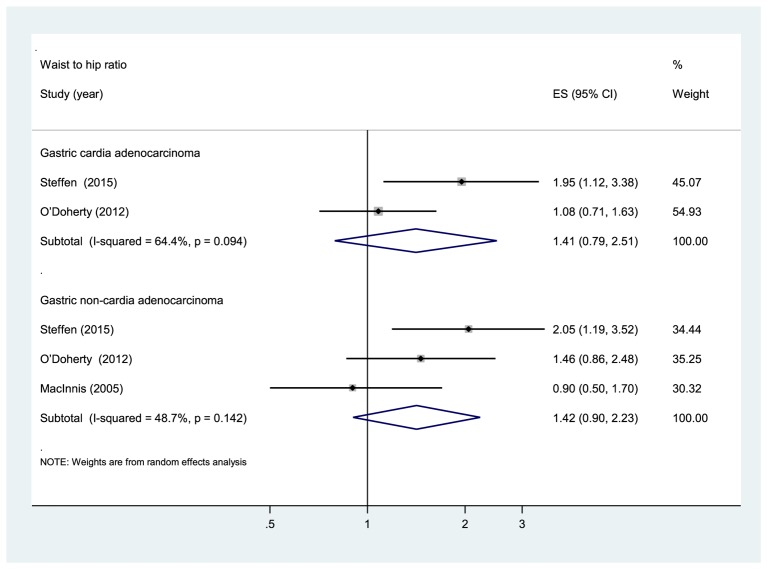
Subgroup analyses according to anatomic subtypes of GC (WHR).

Five prospective cohort studies [24–27,29] were eligible for the analysis of WHR. Comparison of the highest category of WHR with the lowest category revealed significant associations between higher WHR and increased risk of total gastroesophageal cancer (RR: 1.49, 95% CI: 1.19, 1.88), GC (RR: 1.33, 95% CI: 1.04, 1.70), and esophageal cancer (RR: 1.99, 95% CI: 1.05, 3.75) (Figure 4). The evidence of moderate heterogeneity were observed for total gastroesophageal cancer (*I*^2^ =44.2%, *P*=0.064), as well as for GC (*I*^2^ =35.9%, *P*=0.167), while high heterogeneity was observed for esophageal cancer (*I*^2^ =62.2%, *P*=0.071). Regarding the anatomic subtypes of GC, no significant association was found between WHR and adenocarcinomas of gastric cardia (RR: 1.41, 95% CI: 0.79, 2.51; *I*^2^ =64.4%, *P*=0.094) and gastric non-cardia (RR: 1.42, 95% CI: 0.90, 2.23; *I*^2^ =48.7%, *P*=0.142) (Figure 5). In analysis restricted to studies that adjusted for BMI [25,27,29], higher WHR was positively associated with GC (RR: 1.40, 95% CI: 1.08, 1.82; *I*^2^ =36%, *P*=0.181), whereas no association was found with esophageal cancer (RR: 2.30, 95% CI: 0.86, 6.17; *I*^2^ =80.5%, *P*=0.024). The sensitivity analyses omitting one study at a time and calculating the combined RRs for the remaining studies showed that the combined RRs was not substantially affected by any single study. No evidence of publication bias was observed across studies (Begg, *P*>0.1; Egger, *P*>0.1).

## Discussion

The present comprehensive systematic review and meta-analysis examined the associations between abdominal obesity and risk of total gastroesophageal cancer, GC and esophageal cancer. We found evidence of an increased risk of total gastroesophageal cancer, GC, and esophageal cancer with higher WC and WHR.

To the best of our knowledge, this is the first systematic review and meta-analysis to summarise the available evidence from prospective studies for determining the associations between abdominal obesity and risk of total gastroesophageal cancer and GC. We are fully aware of previous meta-analysis of central adiposity and esophageal cancer [42]. However, this meta-analysis used the combined results from case-control and prospective cohort studies together in the same analysis. Such practice is not acceptable within modern meta-analyses due to the different robustness. Case-control studies are more prone to recall bias and selection bias, and typically give different results to the cohort studies. The authors also combined RRs of all different central adiposity measures (WC, WHR, and abdominal diameter) together in one analysis, rather than separately. In contrast with previous meta-analysis, our meta-analysis analyzed the associations between abdominal adiposity measures and esophageal cancer separately and included one more prospective cohort study [26]. In addition, the updated findings [27] from the European Prospective Investigation into Cancer and Nutrition (EPIC) study were used instead of the previous one [35].

Findings on the associations between measures of abdominal obesity and anatomic subtypes of GC in prospective studies have been inconsistent. MacInnis et al. [24] found that higher WC, but not WHR, was associated with an increased risk of adenocarcinoma of lower esophagus and gastric cardia. In the present study, no association was found between these adiposity measures and GNCA [24]. The prospective NIH-AARP (National Institutes of Health-American Association of Retired Persons) observed a significantly higher risk of GCA with WC. WHR was only associated with GCA in the age- and sex-adjusted model, but was attenuated after multivariate adjustment. In contrast, both abdominal adiposity measures were not associated with GNCA [25]. Furthermore, the EPIC reported significant positive associations between WHR and adenocarcinomas of gastric cardia and gastric non-cardia, whereas WC was related to GCA only [27]. Findings from our subgroup analyses showed non-significant positive associations between GNCA and both measures of abdominal adiposity, while GCA was positively associated with WC but not with WHR. Nevertheless, WC and WHR provided only crude measures of intra-abdominal fat. Therefore, future studies with advanced imaging techniques (i.e. magnetic resonance imaging and computed tomography) are warranted to confirm these findings.

As mentioned earlier, obesity has been shown to be an important risk factor for certain types of cancer [2–7]. However, most researchers put more effort to examine the role of general obesity in the etiologies of these malignancies, while paying less attention to the potential detrimental effect of abdominal obesity. Likewise, it remains unclear whether the effect of abdominal obesity on gastric and esophageal cancers is independent of general obesity. In our pooled analysis restricted to studies that further adjusted for BMI, WC was positively associated with GC and esophageal cancer, whereas WHR was positively associated with risk of GC only and not with esophageal cancer. These findings were somewhat surprising since esophagus is located adjacent to stomach, and both conditions are thought to share common risk factors. In the meantime, currently available evidence is too sparse to draw reliable conclusions on BMI-independent effect of central obesity on these malignancies. Further assessment in any future studies is needed to address this discrepancy.

The link between esophageal adenocarcinoma is notably stronger for abdominal obesity than general obesity [43]. A potential mechanical mechanism linking abdominal obesity with EAC is via gastroesophageal reflux disease (GERD) due to enhanced intra-abdominal pressure and the lower esophageal sphincter predisposing to Barrett’s esophagus and finally leading to EAC [44–47]. Several experimental studies have showed that acids and bile acids may promote esophageal carcinogenesis by increasing proliferation, inhibiting apoptosis and generating free radicals [48–50]. On the basis of these findings, it is expected that amongst GERD patients, those taking acid-suppression medications would have lower risk of EAC than those not taking such medications. Conversely, this hypothesis does not appear to be supported by previous studies which reporting lack of association between acid suppression medications and GERD-induced EAC [51,52]. The association between GERD and esophageal cancer is relatively clear, but less so for gastric cardia carcinoma [9]. Although adenocarcinoma of the gastric cardia is more prevalent than EAC [53,54], the mechanisms underlying GCA carcinogenesis has been understudied. However, previous study on events at gastric cardia may provide additional explanation for the pathogenesis of EAC and GCA [55]. A group of middle-aged participants without *H. pylori* infection and evidence of traditional reflux disease were enrolled in the present study to determine the associations between central obesity and the length of cardiac mucosa, inflammation of the distal segment of the lower esophageal sphincter, pH, and other measurements of gastroesophageal function. In the present study, inflammatory cells were detected at the gastric cardia in all participants, but those with large WC and higher total abdominal fat were found to exhibit greater cardiac mucosal lengthening and had more proximal acid reflux. Gastric mucosal lengthening may trigger the expansion and outward migration of cardia progenitor cells into the gastroesophageal junction, leading to gastroesophageal junction neoplasia [56].

Abdominal obesity may affect the gastroesophageal junction not only mechanically but also systemically via metabolic/inflammatory pathways. One plausible molecular mechanism for obesity-associated carcinogenesis is that visceral adipose tissue, which is metabolically active, promotes the release of inflammatory cytokines and mediators, including free fatty acids, tumor necrosis factor α (TNFα), leptin, and resistin, inhibits the secretion of adiponectin and ultimately leads to development of insulin resistance [8,9,57]. In obesity, insulin resistance leads to chronic hyperinsulinemia. Elevated levels of circulating insulin promote carcinogenesis partly by stimulating the production of insulin-like growth factor (IGF-1) and it also inhibits production of IGF-binding proteins (IGFBP-1) 1 and 3 (IGFBP-3). These endocrine disruptions led to an increase in circulating IGF-1, which can bind to the IGF receptor complex, and correspondingly activate pathways that stimulate cell proliferation and impair apoptosis, leading to tumor cell growth [58]. Furthermore, serum levels of IGF-1 have been reported to be higher in EAC patients and in viscerally obese patients [59,60]. Lower plasma adiponectin levels and higher levels of IGF-1 were also observed in patients with upper GC compared with healthy controls [61,62].

## Strengths and limitations

This meta-analysis has several strengths, including incorporated evidence and relevant studies to the date. Because results from individual studies had insufficient statistical power, the enlarged sample size from our meta-analysis may enhance the power to detect a significant association and provide more precise estimates of the effects. All original studies included are of long follow-up durations, and used a prospective design which thereby reduced the likelihood of potential biases (i.e. recall and selection biases). Finally, the prospective design permits the evaluation abdominal adiposity measures before the weight loss that often accompanies gastroesophageal malignancies.

There are several limitations in the present meta-analysis that should be acknowledged. First, heterogeneity was observed across studies, this may attribute to differences in the strength of the association, rather than due to differences in the directionality of effect as all studies showed a clear trend toward increased risk. The observed heterogeneity could also be explained by differences in duration of follow-up, sample sizes, population characteristics, and statistical adjustments for potential confounders. Second, data from original studies were insufficient to evaluate a dose–response relationship since most of included studies did not reported cut-off points for every quantiles of abdominal adiposity measures. Although contacting authors was an option to overcome this problem, however, our approach to overcome this issue was not successful. Third, only seven studies were eligible for this meta-analysis. One of them was conducted in participants with Barrett’s esophagus [26], which may limit the generalisation of the findings. Fourth, although our analysis suggest that both high WC and WHR increase gastric and esophageal cancers risk, few studies have conducted further adjustments between these measures and BMI to try to clarify their independent role. Fifth, only one study has examined the histologic subtypes of esophageal cancer separately [28]. In the present study, high WC was positively associated with risk of EAC but not with esophageal squamous cell carcinoma. Finally, although individual studies have considered a wide range of potential confounders in their analyses, the potential affects of residual/unknown confounding factors on our findings cannot be completely excluded. Given all these limitations, results from our meta-analysis should always be interpreted with caution.

## Conclusion

Although limited, the findings from our meta-analysis suggest the potential role of abdominal obesity in the etiology of gastric and esophageal cancers. The findings from our present meta-analysis highlight the importance of maintaining a healthy weight and staying as lean as possible for reducing risk of gastric and esophageal cancers. Most current evidence suggest mechanical forces caused by increased abdominal pressure and/or endocrinological changes due to excess abdominal adipose tissue as potential risk factors for these cancers. Nevertheless, further research on the mechanisms linking abdominal obesity to adenocarcinoma of esophagus and gastric cardia is warranted to elucidate the exact pathogenic pathways underlying these malignancies and thereby develop better treatment strategies. Furthermore, it will be important for the future prospective studies to examine the potential influences of general obesity (BMI) on these associations, and to provide results according to histologic subtypes. Finally, there are several risk factors (e.g., smoking, physical inactivity, poor diet) common to both abdominal obesity and cancer; given this consideration, the observed association between abdominal obesity and gastroesophageal cancer may be partly due to similar factors shared by both conditions. Thus, further clarification for the issue of whether abdominal obesity itself, rather than a proxy for another cancer risk factor, associated with increased risk of gastroesophageal cancer is warranted.

## Abbreviations

BMI: body mass index
CI: confidence interval
EAC: esophageal adenocarcinoma
EPIC: European Prospective Investigation into Cancer and Nutrition
GC: gastric cancer
GCA: gastric cardia adenocarcinoma
GERD: gastroesophageal reflux disease
GNCA: gastric non-cardia adenocarcinoma
IGF: Insulin-like growth factor
IGFBP: IGF-binding proteins
NIH-AARP: National Institutes of Health-American Association of Retired Persons
NOS: Newcastle-Ottawa Scale
NSAIDs: non-steroidal anti-inflammatory drugs
OR: odds ratio
Q: quantile
RR: relative risk
WC: waist circumference
WHR: waist to hip ratio
